# Targeting 4-1BB for tumor immunotherapy from bench to bedside

**DOI:** 10.3389/fimmu.2022.975926

**Published:** 2022-09-16

**Authors:** Ya-Tao Wang, Wei-Dong Ji, Hong-Mei Jiao, Ang Lu, Kun-Feng Chen, Qi-Bing Liu

**Affiliations:** ^1^ First People’s Hospital of Shangqiu, Henan Province, Shangqiu, China; ^2^ Department of Pharmacy, the First Affiliated Hospital of Hainan Medical University, Haikou, China; ^3^ Department of Pharmacology, School of Basic Medicine and Life Sciences, Hainan Medical University, Haikou, China

**Keywords:** 4-1BB, immunotherapy, cancer, immune checkpoint inhibitor, clinical trials

## Abstract

Immune dysfunction has been proposed as a factor that may contribute to disease progression. Emerging evidence suggests that immunotherapy aims to abolish cancer progression by modulating the balance of the tumor microenvironment. 4-1BB (also known as CD137 and TNFRS9), a member of tumor necrosis factor receptor superfamily, has been validated as an extremely attractive and promising target for immunotherapy due to the upregulated expression in the tumor environment and its involvement in tumor progression. More importantly, 4-1BB-based immunotherapy approaches have manifested powerful antitumor effects in clinical trials targeting 4-1BB alone or in combination with other immune checkpoints. In this review, we will summarize the structure and expression of 4-1BB and its ligand, discuss the role of 4-1BB in the microenvironment and tumor progression, and update the development of drugs targeting 4-1BB. The purpose of the review is to furnish a comprehensive overview of the potential of 4-1BB as an immunotherapeutic target and to discuss recent advances and prospects for 4-1BB in cancer therapy.

## Introduction

Tumor immunotherapy exerts antitumor efficacy through the interaction of the host immune system with tumor-associated antigens (1). It can restore or enhance the body’s immune system’s natural defenses against tumors, which typically targets specific biomolecules on the surface of cancer cells, exemplified by tumor-associated antigens (2). Immunotherapy including immune checkpoint inhibitor (ICI) and CAR-T therapy has made breakthroughs in tumor treatment, but the overall response rate is not high, and many patients cannot benefit from it (3–6). Therefore, the development of new immune checkpoints and biomarkers and expansion of the beneficiary population from immunotherapy are urgent problems to be solved.

Neoantigen epitopes generated by somatic mutations in cancer cells play an important role in T-cell immune responses, which have become an important driver of immune checkpoint discovery in immunotherapy. 4-1BB, also termed 4-1BB and TNFRSF9, was identified in 1989 and originally described as an inducible gene, which was expressed in T lymphocytes (7). 4-1BB exhibited an important effect in various cells and participated in the activation of multiple immune cells, such as CD8 T cells and cytotoxic T lymphocytes (CTL) (8). Emerging evidence has demonstrated that targeting 4-1BB is a uniquely attractive strategy for tumor immunotherapy (9–13). In this review, we discuss the recent advances and prospects of the cancer immunotherapy checkpoint 4-1BB from the aspects of structure, expression, role in tumor microenvironment, development of clinical drugs targeting 4-1BB, and their combination with traditional treatment methods.

## Structure of 4-1BB and its ligand

4-1BB, a glycosylated type I membrane protein, contains four cysteine-rich pseudo repeats, which contribute to the formation of a cytoplasmic signaling domain, extracellular domain, and short helical transmembrane domain (7). An elongated structure was generally formed by the extracellular domain of TNFR (variation range: 1 to 4 CRDs). Based on this, antibodies can bind to these molecules through many modalities. Efficient binding of 4-1BB L to 4-1BB results in rapid receptor activation in response to antigenic stimulation. 4-1BBL (TNFSF9), a type II membrane protein of the TNF ligand superfamily, is the binding partner of 4-1BB (14, 15). TNFSF members, typically expressed on the cell membrane, exist in a homotrimeric complex (16–18), which can be divided into three parts: (a) LTα, TNF, RANKL, LIGHT, Apo2L/TRAIL, and CD40L (19, 20); (b) BAFF, APRIL, and EDA; and (c) GITRL, 4-1BBL, and OX40L, among which OX40L and GITRL exhibit a flatter conformation (19, 21). The sequences of 4-1BBL were poorly conserved in human and mouse.

As a member of the tumor necrosis factor superfamily, 4-1BB is mostly expressed on the surface of activated T cells but also on B cells, NK cells, and DC cells (22, 23). 4-1BB is widely distributed on various tumor cells (such as lung tumor cells, and leukemia cells) and has been identified in tissues (such as liver cancer tissue, and tumor vessel walls). Alfaro et al. found that 4-1BB is also expressed in tonsil and lymph node follicular structures. Thence, a comprehensive analysis of its distribution helps uncover potential roles and functions.

## Role of 4-1BB in the tumor microenvironment

**Figure 1:**
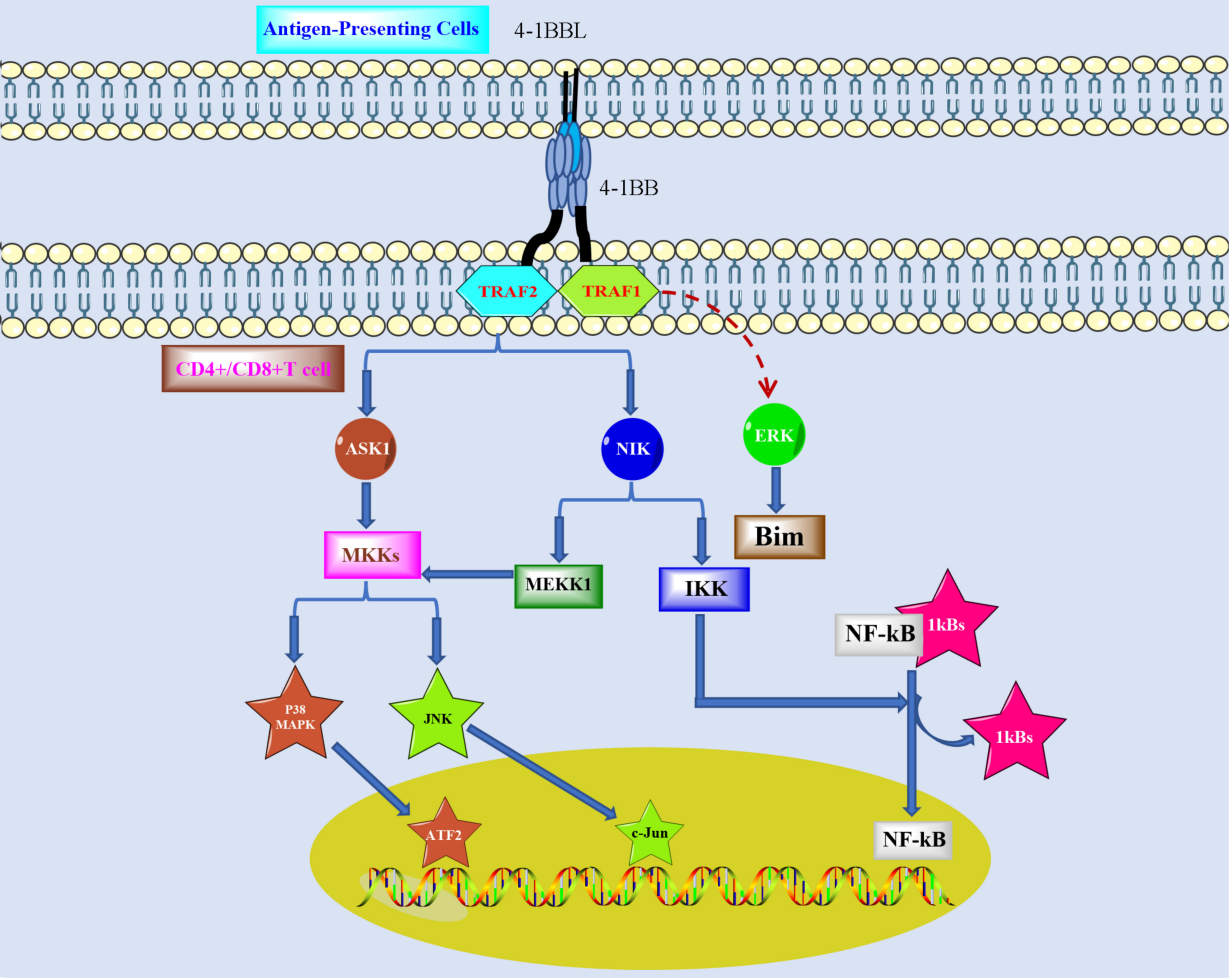
Role of 4-1BB in the tumor microenvironment.

As shown in **Figure 1**, both IL-15 and IL-2 can promote the expression of 4-1BB on NK cells, which stimulates the proliferation of NK cells and produces IFN-γ, thus leading to the activation of T cells (24). 4-1BB facilitates the proliferation of CD8^+^ T cells to produce memory T (Tm) cells (25, 26). Stimulation by 4-1BB will upregulate the expression IL-2 and IFN-γ in CD4^+^ and CD8^+^ T cells. However, 4-1BB expresses a controversial effect in T regulatory cells (Treg), which leads to Treg proliferation but alters Treg for cytotoxic or helper effects (27, 28). 4-1BBL inhibits the conversion of CD4^+^FOXP3^-^ cells to CD4^+^FOXP^+^ (29). 4-1BB is also expressed in monocytes, and it promotes upregulation of IL-8 and TNF-*α* but downregulation of IL-10. The differentiation of monocytes into dendritic cells can be promoted by 4-1BB, and dendritic cells then secrete IL-6 and IL-12 (30). However, 4-1BB stimulation differentiates monocytes into M2 macrophages and accelerates B-cell apoptosis, which also promotes the expression of TNF-*α*/*β* in B cells (31).

## 4-1BB in cancer progression

Through the PI3K/AKT/mTOR pathway, expression of 4-1BB was induced by EBV protein LMP1 to facilitate immune evasion in Hodgkin and Reed–Sternberg cells (32). Low levels of the soluble form of 4-1BBL in patients with AML were associated with better prognosis, especially longer disease-free survival (33). 4-1BB L and 4-1BB were abnormally expressed in tumor cells in hematopoietic malignancies, and their interaction promotes tumor growth in cutaneous T-cell lymphoma (34). Overexpression of 4-1BB on leukemic cells was significantly related to poor prognosis (35). Antitumor activity was enhanced in 4-1BB-knockout mice (36). Similarly, the tumor growth was seriously blocked in 4-1BB knockout mice subcutaneously injected with CT26 cells (37). The findings further proved the critical role of 4-1BB-4-1BBL in tumor development.

## 4-1BB-targeted drug development

The efficacy of the 4-1BB antibody in preventing cancer in animals has prompted clinical development. The use of monoclonal antibodies to treat cancer has achieved great success over the past few decades, many of which have been under evaluation in different clinical trials, as shown in **Table 1**.

**Table 1:** 4-1BB modulators in clinical trials.

Drug	Study Title	ClinicalTrials	Phase	Status
EU 101	A Study to Evaluate Safety, Efficacy, and Pharmacokinetics in Participants With Advanced Solid Tumors	NCT04903873	Phase 1 Phase 2	Recruiting
	Expanded Access Program Using IMM-101 for Patients With Advanced Pancreatic Cancer	NCT04137822	Unknown	No longer available
	A Study of Belinostat + Carboplatin or Paclitaxel or Both in Patients With Ovarian Cancer in Need of Relapse Treatment	NCT00421889	Phase 1 Phase 2	Completed
	Study of Lanreotide in Metastatic or Recurrent Grade I-II Hindgut NET	NCT03083210	Phase 4	Unknown
Urelumab	Urelumab (4-1BB mAb) With Rituximab for Relapsed, Refractory or High-risk Untreated Chronic Lymphocytic Leukemia (CLL) Patients	NCT02420938	Phase 2	Withdrawn
	Combination Study of Urelumab and Rituximab in Patients With B-cell Non-Hodgkins Lymphoma	NCT01775631	Phase 1	Completed
	Phase I-II Study of Intratumoral Urelumab Combined With Nivolumab in Patients With Solid Tumors	NCT03792724	Phase 1 Phase 2	Not yet recruiting
	Combination Study of Urelumab and Cetuximab in Patients With Advanced/Metastatic Colorectal Cancer or Advanced/Metastatic Head and Neck Cancer	NCT02110082	Phase 1	Completed
	Neoadjuvant Nivolumab With and Without Urelumab in Cisplatin-Ineligible or Chemotherapy-refusing Patients With Muscle-Invasive Urothelial Carcinoma of the Bladder	NCT02845323	Phase 2	Recruiting
	An Investigational Immuno-therapy Study to Determine the Safety of Urelumab Given in Combination With Nivolumab in Solid Tumors and B-cell Non-Hodgkin’s Lymphoma	NCT02253992	Phase 1 Phase 2	Terminated
	A Phase I Open Label Study of the Safety and Tolerability of Elotuzumab (BMS-901608) Administered in Combination With Either Lirilumab (BMS-986015) or Urelumab (BMS-663513) in Subjects With Multiple Myeloma	NCT02252263	Phase 1	Completed
	Safety, Tolerability, Pharmacokinetics, and Immunoregulatory Study of Urelumab (BMS-663513) in Subjects With Advanced and/or Metastatic Solid Tumors and Relapsed/Refractory B-cell Non-Hodgkin’s Lymphoma	NCT01471210	Phase 1	Completed
	Study of Urelumab in Subjects With Advanced and/or Metastatic Malignant Tumors	NCT02534506	Phase 1	Completed
	A Study of BMS-663513 Administered in Combination With Chemotherapy to Subjects With Advanced Solid Malignancies	NCT00351325	Phase 1	Terminated
	A Study of BMS-663513 in Combination With Chemoradiation in Subjects With Non Small Cell Lung Carcinoma (NSCLC)	NCT00461110	Phase 1	Terminated
	Study of BMS-663513 in Patients With Advanced Cancer	NCT00309023	Phase 1 Phase 2	Terminated
	Stereotactic Body Radiotherapy (SBRT) Plus Immunotherapy for Cancer	NCT03431948	Phase 1	Active, not recruiting
	Anti-LAG-3 Alone and in Combination w/Nivolumab Treating Patients w/Recurrent GBM (Anti-4-1BB Arm Closed 10/16/18)	NCT02658981	Phase 1	Active, not recruiting
	Phase II, 2nd Line Melanoma - RAND Monotherapy	NCT00612664	Phase 2	Completed
	Combination of Anti-4-1BB and Ipilimumab in Patients With Melanoma	NCT00803374	Phase 1	Withdrawn
	Platform Study of Neoadjuvant and Adjuvant Immunotherapy for Patients With Resectable Adenocarcinoma of the Pancreas	NCT02451982	Phase 2	Recruiting
	Combining PD-1 Blockade, 4-1BB Agonism and Adoptive Cell Therapy for Metastatic Melanoma	NCT02652455	Early Phase 1	Active, not recruiting
Sytalizumab	The Safety and Efficacy of TWP-101 in Patients With Advanced Solid Tumor	NCT04871347	Phase 1	Not yet recruiting
	Safety, Tolerability and Pharmacokinetics of TWP-101 in Patients With Advanced Melanoma and Urothelial Carcinoma	NCT04871334	Phase 1	Recruiting
LVGN-6051	A Study of LVGN6051 Combined With Anlotinib in Patient With Soft Tissue Sarcoma	NCT05301764	Phase 1 Phase 2	Recruiting
	Phase 1 Trial of LVGN6051 as Single Agent and in Combination With Keytruda (MK-3475-A31/KEYNOTE-A31) in Advanced or Metastatic Malignancy	NCT04130542	Phase 1	Recruiting
	Study of LVGN6051 (4-1BB Agonist Antibody) in Advanced or Metastatic Malignancy	NCT04694781	Phase 1	Recruiting
	Study of LVGN3616 and LVGN6051 ± LVGN7409 in Combination With Nab-Paclitaxel or Bevacizumab and Cyclophosphamide in Metastatic Solid Tumors	NCT05075993	Phase 1	Recruiting
	Phase 1 Trial of LVGN7409 (CD40 Agonist Antibody) as Single Agent and Combination Therapies in Advanced or Metastatic Malignancy	NCT04635995	Phase 1	Recruiting
YH-004	Study of YH004 (4-1BB Agonist Antibody) in Advanced or Metastatic Malignancy	NCT05040932	Phase 1	Recruiting
GEN1046	GEN1046 Safety and PK in Subjects With Advanced Solid Malignancies	NCT04937153	Phase 1	Recruiting
	Safety and Efficacy Study of GEN1046 as a Single Agent or in Combination With Another Anti-cancer Therapy for Treatment of Recurrent (Non-small Cell) Lung Cancer	NCT05117242	Phase 2	Recruiting
	GEN1046 Safety Trial in Patients With Malignant Solid Tumors	NCT03917381	Phase 1 Phase 2	Recruiting
PRS343	PRS-343 in HER2-Positive Solid Tumors	NCT03330561	Phase 1	Completed
	PRS-343 in Combination With Atezolizumab in HER2-Positive Solid Tumors	NCT03650348	Phase 1	Active, not recruiting
	Cinrebafusp Alfa in Combination With Ramucirumab and Paclitaxel in HER2-High Gastric or GEJ Adenocarcinoma and in Combination With Tucatinib in HER2-Low Gastric or GEJ Andenocarinoma	NCT05190445	Phase 2	Recruiting
ES101	A Study of ES101 (PD-L1x4-1BB Bispecific Antibody) in Patients With Advanced Malignant Thoracic Tumors	NCT04841538	Phase 1 Phase 2	Withdrawn
	A Study of ES101 (PD-L1x4-1BB Bispecific Antibody) in Patients With Advanced Solid Tumors	NCT04009460	Phase 1	Terminated
	Ankle - Brachial Index Measurement in Atrial Fibrillation	NCT02986282	Not applicable	Completed
Cinrebafusp alfa	Cinrebafusp Alfa in Combination With Ramucirumab and Paclitaxel in HER2-High Gastric or GEJ Adenocarcinoma and in Combination With Tucatinib in HER2-Low Gastric or GEJ Andenocarinoma	NCT05190445	Phase 2	Recruiting
HLX-35	HLX35(EGFR×4-1BB Bispecific) in Patients With Advanced or Metastatic Solid Tumors	NCT05360381	Phase 1	Not yet recruiting
IBI319	Study of the Efficacy and Safety of IBI319 in Patients With Advanced Malignant Tumors	NCT04708210	Phase 1	Recruiting
TJ-033721	Study of TJ033721 in Subjects With Advanced or Metastatic Solid Tumors	NCT04900818	Phase 1	Recruiting
ATG 101	A Study of Evaluating the Safety and Efficacy of ATG-101 in Patients With Metastatic/Advanced Solid Tumors and Mature B-cell Non-Hodgkin Lymphomas	NCT04986865	Phase 1	Recruiting
	Study of ASC-101 in Patients With Hematologic Malignancies Who Receive Dual-cord Umbilical Cord Blood Transplantation	NCT01983761	Phase 1 Phase 2	Recruiting
	Safety and Efficacy of Two Doses of ATIR101, a T-lymphocyte Enriched Leukocyte Preparation Depleted of Host Alloreactive T-cells, in Patients With a Hematologic Malignancy Who Received a Hematopoietic Stem Cell Transplantation From a Haploidentical Donor	NCT02500550	Phase 2	Completed
	Antithymocyte Globulin and Cyclosporine in Preventing Graft-Versus-Host Disease in Patients Undergoing Chemotherapy With or Without Radiation Therapy Followed By Donor Stem Cell Transplant for Acute Lymphoblastic Leukemia or Acute Myeloid Leukemia	NCT00093587	Not applicable	Unknown
	Thymoglobulin to Prevent Acute Graft vs. Host Disease (GvHD) in Patients With Acute Lymphocytic Leukemia (ALL) or Acute Myelogenous Leukemia (AML) Receiving a Stem Cell Transplant	NCT00088543	Not applicable	Completed
LBL-024	A Phase I/II Clinical Study of LBL-024 in Patients With Advanced Malignant Tumors	NCT05170958	Phase 1 Phase 2	Recruiting
MCLA-145	A Study of Bispecific Antibody MCLA-145 in Patients With Advanced or Metastatic Malignancies	NCT03922204	Phase 1	Recruiting
ABL-503	This is a Study to Evaluate the Safety and Tolerability of ABL503, and to Determine the Maximum Tolerated Dose (MTD) and Recommended Phase 2 Dose (RP2D) of ABL503 in Subjects With Any Progressive Locally Advanced or Metastatic Solid Tumors	NCT04762641	Phase 1	Recruiting
PM 1032	A Study of Ramucirumab (IMC-1121B) and Paclitaxel in Participants With Solid Tumors	NCT01515306	Phase 2	Completed
QLF-31907	A Phase Ia Clinical Study of QLF31907 Injection in Patients With Advanced Malignant Tumors	NCT05150405	Phase 1	Recruiting
FS-120	FS120 First in Human Study in Patients With Advanced Malignancies	NCT04648202	Phase 1	Recruiting
RO-7227166	A Study to Evaluate the Safety, Pharmacokinetics and Preliminary Anti-Tumor Activity of RO7227166 in Combination With Obinutuzumab and in Combination With Glofitamab Following a Pre-Treatment Dose of Obinutuzumab Administered in Participants With Relapsed/Refractory B-Cell Non-Hodgkin’s Lymphoma	NCT04077723	Phase 1	Recruiting
HBM-7008	HBM7008 -Study on Subjects With Advanced Solid Tumors	NCT05306444	Phase 1	Recruiting
ND-021	A Study of NM21-1480 in Adult Patients With Advanced Solid Tumors	NCT04442126	Phase 1 Phase 2	Recruiting
GNC-035	A Study of GNC-035, a Tetra-specific Antibody, in Participants With Locally Advanced or Metastatic Breast Cancer	NCT05160545	Phase 1	Recruiting
	A Study of GNC-035, a Tetra-specific Antibody, in Participants With Locally Advanced or Metastatic Solid Tumors	NCT05039931	Phase 1	Recruiting
	A Study of GNC-035, a Tetra-specific Antibody, in Participants With Relapsed/Refractory Hematologic Malignancy	NCT05104775	Phase 1	Recruiting
GNC-038	A Study of GNC-038, a Tetra-specific Antibody, in Participants With R/R Diffuse Large B-cell Lymphoma (DLBCL)	NCT05192486	Phase 1 Phase 2	Recruiting
	A Study of GNC-038, a Tetra-specific Antibody, in Participants With R/R Non-Hodgkin Lymphoma	NCT04606433	Phase 1	Recruiting
	Mechanism of Resistance to GNC-038 in Relapsed and Refractory Diffuse Large B-cell Lymphoma	NCT05189782	Unknown	Recruiting
GNC-039	A Study of GNC-039, a Tetra-specific Antibody, in Participants With Relapsed/Refractory or Metastatic Solid Tumors	NCT04794972	Phase 1	Recruiting
ADG-106	A Study to Evaluate the Combination of Nivolumab With ADG106 in Metastatic NSCLC	NCT05236608	Phase 1 Phase 2	Recruiting
	Study of ADG106 In Combination With PD-1 Antibody In Advanced or Metastatic Solid Tumors and/or Non Hodgkin Lymphoma	NCT04775680	Phase 1 Phase 2	Recruiting
	A Phase Ib Safety lead-in, Followed by Phase II Trial of ADG106 in Combination With Neoadjuvant Chemotherapy in HER2 Negative Breast Cancer	NCT05275777	Phase 1 Phase 2	Recruiting
	Study of ADG106 With Advanced or Metastatic Solid Tumors and/or Non-Hodgkin Lymphoma	NCT03802955	Phase 1	Active, not recruiting
	Study of 4-1BB Agonist ADG106 With Advanced or Metastatic Solid Tumors and/or Non-Hodgkin Lymphoma	NCT03707093	Phase 1	Active, not recruiting
	ADG126, ADG126 in Combination With Anti PD1 Antibody, and ADG126 in Combination With ADG106 in Advanced/Metastatic Solid Tumors	NCT04645069	Phase 1	Recruiting
	A Phase 1b Study of ADG116, ADG116 Combined With Anti-PD-1 Antibody or Anti-4-1BB Antibody in Solid Tumors Patients	NCT04501276	Phase 1	Recruiting
Utomilumab	Utomilumab and ISA101b Vaccination in Patients With HPV-16-Positive Incurable Oropharyngeal Cancer	NCT03258008	Phase 2	Completed
	T-Cell Infusion, Aldesleukin, and Utomilumab in Treating Patients With Recurrent Ovarian Cancer	NCT03318900	Phase 1	Active, not recruiting
	Safety and Efficacy of Axicabtagene Ciloleucel in Combination With Utomilumab in Adults With Refractory Large B-cell Lymphoma	NCT03704298	Phase 1	Active, not recruiting
	Avelumab, Utomilumab, Rituximab, Ibrutinib, and Combination Chemotherapy in Treating Patients With Relapsed or Refractory Diffuse Large B-Cell Lymphoma or Mantle Cell Lymphoma	NCT03440567	Phase 1	Active, not recruiting
	The AVIATOR Study: Trastuzumab and Vinorelbine With Avelumab OR Avelumab and Utomilumab in Advanced HER2+ Breast Cancer	NCT03414658	Phase 2	Recruiting
	4-1BB Agonist Monoclonal Antibody PF-05082566 With Trastuzumab Emtansine or Trastuzumab in Treating Patients With Advanced HER2-Positive Breast Cancer	NCT03364348	Phase 1	Active, not recruiting
	Utomilumab, Cetuximab, and Irinotecan Hydrochloride in Treating Patients With Metastatic Colorectal Cancer	NCT03290937	Phase 1	Active, not recruiting
	Avelumab, Utomilumab, Anti-OX40 Antibody PF-04518600, and Radiation Therapy in Treating Patients With Advanced Malignancies	NCT03217747	Phase 1 Phase 2	Active, not recruiting
	RITUXIMAB + IMMUNOTHERAPY IN FOLLICULAR LYMPHOMA	NCT03636503	Phase 1	Active, not recruiting
	A Study Of Avelumab In Combination With Other Cancer Immunotherapies In Advanced Malignancies (JAVELIN Medley)	NCT02554812	Phase 2	Active, not recruiting
	Avelumab In Combination Regimens That Include An Immune Agonist, Epigenetic Modulator, CD20 Antagonist and/or Conventional Chemotherapy in Patients With Relapsed or Refractory Diffuse Large B-cell Lymphoma (R/R DLBCL)	NCT02951156	Phase 3	Terminated
	Avelumab With Binimetinib, Sacituzumab Govitecan, or Liposomal Doxorubicin in Treating Patients With Stage IV or Unresectable, Recurrent Triple Negative Breast Cancer	NCT03971409	Phase 2	Recruiting
	Continued Access Study for Participants Deriving Benefit in Pfizer-Sponsored Avelumab Parent Studies That Are Closing	NCT05059522	Phase 3	Recruiting
	Study Of OX40 Agonist PF-04518600 Alone And In Combination With 4-1BB Agonist PF-05082566	NCT02315066	Phase 1	Completed
ATOR-1017	ATOR-1017 First-in-human Study	NCT04144842	Phase 1	Recruiting
AGEN-2373	Anti-4-1BB and Anti-CTLA-4 Monoclonal Antibody in Patient With Advanced Cancer	NCT04121676	Phase 1	Recruiting
CTX-471	Study of CTX-471 in Patients Post PD-1/PD-L1 Inhibitors in Metastatic or Locally Advanced Malignancies	NCT03881488	Phase 1	Recruiting
PRS-344	A Study of PRS-344/S095012 (PD-L1x4-1BB Bispecific Antibody-Anticalin Fusion) in Patients With Solid Tumors	NCT05159388	Phase 1 Phase 2	Recruiting
RO-7122290	Study To Evaluate Safety, Pharmacokinetics, Pharmacodynamics, And Preliminary Anti-Tumor Activity Of RO7122290 In Combination With Cibisatamab With Obinutuzumab Pre-Treatment	NCT04826003	Phase 1 Phase 2	Recruiting
	Study Evaluating the Efficacy and Safety of Multiple Immunotherapy-Based Treatments and Combinations in Patients With Urothelial Carcinoma (MORPHEUS-UC)	NCT03869190	Phase 1 Phase 2	Recruiting
Anti BCMA CART cell therapy	Anti-BCMA or/and Anti-CD19 CART Cells Treatment of Relapsed Multiple Myeloma	NCT03767725	Phase 1	Unknown
	BCMA Chimeric Antigen Receptor Expressing T Cells Therapy for Relapsed/Refractory Multiple Myeloma	NCT03943472	Early Phase 1	Recruiting
	Master Protocol for the Phase 1 Study of Cell Therapies in Multiple Myeloma	NCT04155749	Phase 1	Recruiting
	Study of T Cells Targeting CD19/BCMA (CART-19/BCMA) for High Risk Multiple Myeloma Followed With Auto-HSCT	NCT03455972	Phase 1 Phase 2	Recruiting
	A Study of BCMA-directed CAR-T Cells Treatment in Subjects With r/r Multiple Myeloma	NCT03751293	Phase 1	Unknown
	Clinical Trial Using Humanized CART Directed Against BCMA (ARI0002h) in Patients With Relapsed/Refractory Multiple Myeloma to Proteasome Inhibitors, Immunomodulators and Anti-CD38 Antibody.	NCT04309981	Phase 1 Phase 2	Recruiting
	A Study of BCMA-directed CAR-T Cells Treatment in Subjects With r/r Multiple Myeloma	NCT04322292	Phase 1	Unknown
	BCMA-directed CAR-T Cell Therapy in Adult Patients With Relapsed and/or Refractory Multiple Myeloma	NCT04318327	Phase 1	Recruiting
	Autologous CD8+ T-cells Expressing an Anti-BCMA CAR in Patients With Myeloma	NCT03448978	Phase 1 Phase 2	Completed
	CART-BCMA Cells for Multiple Myeloma	NCT02546167	Phase 1	Completed
	Humanized CAR-T Cells of Anti-BCAM and Anti-CD19 Against Relapsed and Refractory Multiple Myeloma	NCT04194931	Phase 1	Unknown
	BCMA Chimeric Antigen Receptor Expressing T Cells in Multiple Myeloma	NCT03093168	Phase 1	Unknown
	Safety and Efficacy Evaluation of BCMA-CART for Treating Multiple Myeloma	NCT03492268	Not applicable	Withdrawn
	Efficacy and Safety Evaluation of BCMA-UCART	NCT03752541	Not applicable	Suspended
HOT-1030	A Study of HOT1030 in Patients With Advanced Solid Tumors	NCT05060263	Phase 1	Recruiting
Delolimogene mupadenorepvec	A Phase I/II Trial Investigating LOAd703 in Combination With Atezolizumab in Malignant Melanoma	NCT04123470	Phase 1 Phase 2	Recruiting
	A Study Evaluating the Efficacy and Safety of Multiple Immunotherapy-Based Treatment Combinations in Patients With Metastatic Colorectal Cancer (Morpheus-CRC)	NCT03555149	Phase 1 Phase 2	Recruiting
	LOAd703 Oncolytic Virus Therapy for Pancreatic Cancer	NCT02705196	Phase 1 Phase 2	Recruiting
BT 7480	Study BT7480-100 in Patients With Advanced Malignancies Associated With Nectin-4 Expression	NCT05163041	Phase 1 Phase 2	Recruiting

Urelumab (BMS-663513), the first 4-1BB-targeted therapy to enter clinical trials developed by Bristol–Myers Squibb, is a human IgG4 human monoclonal antibody, which will not inhibit the interaction between 4-1BB with its ligand (38). Preliminary clinical results in phase 1/2 disclosed in 2008 showed encouraging efficacy, but further development was hindered by liver toxicity (39). Urelumab reentered clinical trials in 2012, which was combined with nivolumab, cetuximab, rituximab, and elotuzumab, respectively (12). However, hepatotoxicity of the antibody emerged shortly thereafter, causing the urelumab development program to be shelved. Currently, urelumab, a potent agonist mAb, is still under different clinical trials (**Table 1**), and strategies to avoid hepatotoxicity and achieve appropriate drug exposure levels are worth investigating. Utomilumab (PF-05082566) is a 4-1BB-humanized IgG2 monoclonal antibody developed by Pfizer (40). Compared with urelumab, it has a higher safety profile and is currently undergoing multiple clinical trials (41).

To reduce the hepatotoxicity of systemic 4-1BB agonists, the development of bispecific antibodies against 4-1BB has been recognized as a viable strategy, and some bispecific antibodies, including GEN1046 (PD-L1/4-1BB) and PRS343 (HER2/4-1BB), are currently being evaluated in different clinical trials (**Table 1**) (42, 43). ES101 (INBRX-105), a first-in-class tetravalent bispecific antibody targeting PD-L1/4-1BB, originally developed by Inhibrx, was introduced into its Greater China rights by Kewan Pharmaceuticals (44). It contains four domains, and two of them target PD-L1 while the other two target 4-1BB, which can alleviate PD-1/PD-L1-mediated immune checkpoint inhibition. The 4-1BB-binding domain may drive the aggregation of 4-1BB molecules on the surface of T cells, so that 4-1BB-mediated immune activation can be concentrated on T cells near the tumor, effectively reducing the potential off-target toxicity.

In addition to double-antibody drugs, the development of 4-1BB targets has been extended to tertiary and tetraspecific antibodies. NM21-1480 is a monovalent trispecific antibody fragment molecule against PD-L1, 4-1BB, and human serum protein (HSA) (45). NM21-1480 exerts a synergistic effect of 4-1BB agonism and PD-L1 blockade and shows an extended half-life by binding to HSA, thereby reducing the frequency of dosing. GNC-035 is a four-antibody drug targeting PD-L1/CD3/4-1BB/ROR1 while GNC-039 targets PD-L1/4-1BB/CD3/EGFR. In terms of design, both GNC-035 and GNC-039 build symmetrical tetraspecific antibodies based on IgG with three scFvs in series. Among them, PD-L1, 4-1BB, and CD3 are immunoregulatory functions, and the fourth target is tumor antigen. Both drugs are undergoing evaluation in different clinical trials (**Table 1**).

## Future directions

Immunotherapy is known as the fourth cancer treatment after surgery, radiotherapy, and chemotherapy, which has changed the treatment patterns of patients with advanced tumors (46). However, only a minority of cancer patients can benefit from it. Treatment methods such as surgery, chemotherapy, radiotherapy, and targeted therapy can synergize with immunotherapy to enhance the curative effect. Guillerey et al. found that anti-4-1BB mAb combined with chemotherapy could prevent MM relapse and prolong survival in MM mice (47). A study undertaken by Newcomb et al. demonstrated that radiation could synergistically enhance the antitumor effect of anti-4-1BB therapy in a mouse glioma model (48). Moreover, anti-4-1BB mAbs could enhance the efficacy of other antitumor Abs (such as cetuximab, rituximab, and trastuzumab) and exert synergistic effects. Taken together, combination therapy for tumors may also be the future direction of tumor therapy.

## Conclusion

To summarize, existing studies support immunotherapies targeting the 4-1BB pathway for the treatment of cancer. In the study, we have summarized the structure of 4-1BB and its ligand as well as the expression in various immune cells and tumor cells. More importantly, we discuss the role of 4-1BB in the microenvironment and tumor progression. Furthermore, the development of drug-targeted 4-1BB was summarized and updated, which exhibited tremendous potential in clinical trials. Although the anti-4-1BB therapy provides hope for cancer treatment, the effectiveness of drugs targeting 4-1BB in clinical antitumor therapy alone or in combination with other antitumor therapies still needs to be investigated in the future.

## Author contributions

Y-TW, K-FC and Q-BL conceived the review. All authors contributed to the article and approved the submitted version.

## Funding

This work was funded by the National Natural Science Foundation of China (No. 81960663).

## Conflict of interest

The authors declare that the research was conducted in the absence of any commercial or financial relationships that could be construed as a potential conflict of interest.

## Publisher’s note

All claims expressed in this article are solely those of the authors and do not necessarily represent those of their affiliated organizations, or those of the publisher, the editors and the reviewers. Any product that may be evaluated in this article, or claim that may be made by its manufacturer, is not guaranteed or endorsed by the publisher.
